# Rings or daggers, axes or fibulae have a different composition? A multivariate study on Central Italy bronzes from eneolithic to early iron age

**DOI:** 10.1186/s13065-015-0090-7

**Published:** 2015-04-01

**Authors:** Giovanni Visco, Susanne H Plattner, Giuseppe Guida, Stefano Ridolfi, Giovanni E Gigante

**Affiliations:** University “Sapienza”, Piazzale Aldo Moro 5, 00185 Rome, Italy; Central Institute of Restoration – ISCR, Via di San Michele 23, 00153 Rome, Italy; Ars Mensurae, Rome, Italy

**Keywords:** Ancient bronze composition, Chemometrics, Multivariate analysis

## Abstract

**Background:**

One of the main concerns for archaeo-metallurgists and archaeologists is to determine to what extent ancient craftsmen understood the effect of metal alloy composition and were able to control it in order to produce objects with the most suitable features.

This problem can be investigated by combining compositional analyses of a high number of ancient artefacts with correlation analyses of the objects’ age, production site, destination of usage etc. – and thus chemometric data treatment is carried out. In this study, multivariate analyses were performed on a matrix composed of elemental compositional data from 134 archaeological bronze objects, obtained by XRF analyses. Analysed objects have been dated back from the Eneolithic Period to the end of the Bronze Age including the early Iron Age and were excavated in Central Italy (mainly Abruzzo Region).

**Results:**

Chemometric analysis was performed attempting to visualise clouds of objects through PCA. In parallel and independently, object grouping was attempted using several different approaches, based on object characteristics (e.g. shape, weight, type of use – cutting or hitting and age) following indications given by archaeologists (or derived from the archaeological context).

Furthermore, case-tailored data pretreatment (logratio-centred scaling) was used, but no homogeneous groups could be identified.

**Conclusions:**

By using chemometric data analysis, homogeneous groups of objects could not be detected, meaning that compositional data of alloys is not correlated with the considered objects’ characteristics. This favours the conclusion that – without discussing the ascertained ability of ancient foundry-men - they had also already discovered the convenience of recycling broken objects thus producing a more or less similar bronze alloy each time, depending on materials’ availability; necessary mechanical characteristics could then be obtained by post processing.

**Graphical Abstract:**
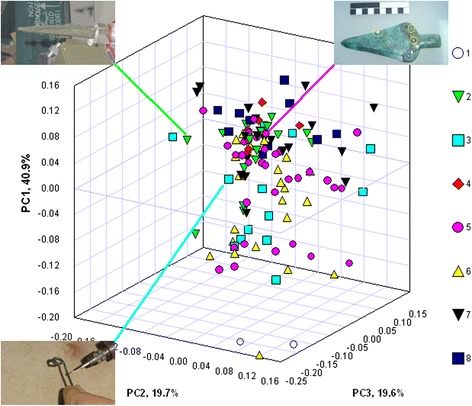
Scores PCA plot in 3D space with 3 different bronze objects.

**Electronic supplementary material:**

The online version of this article (doi:10.1186/s13065-015-0090-7) contains supplementary material, which is available to authorized users.

## Background

A huge number of small bronze fragments can be found in Italy. One of the oldest finds, an axe of pure copper used by Ötzi the Iceman, was found in the Alps of Ötztal, at the boundary between Italy and Austria in 1991 [1]; it dates back to about 3300 b.C. as stated by archaeologists and denotes the overcome of the final Bronze and Copper Ages [2]. Items of copper and its alloys are very common metallic cultural heritage objects. The use of native copper for the production of small pieces of jewelry, weapons and tools by hammering and partial melting, goes way back in time to 7000 b.C. .

Later, similar to nowadays, copper objects were also obtained by smelting ores [3]. In this case, objects generally show characteristic Cu(I) oxide inclusions, formed during the smelting process. The presence of other elements (arsenic, iron, zinc, lead, nickel, antimony, silver) leads us to believe that the production of alloys was probably accidental in the beginning and then later used by metalworkers to influence the colour and other properties (e.g. workability, hardness, etc.) [4] of metal. Further knowledge on this ability of ancient metalworkers is of main concern for metallurgists and archaeologists but the distinction between impure copper artefacts and early bronze ones is not always immediately obvious. In fact, the first copper alloy, copper-arsenic, is thought to have an accidental origin because arsenic is often associated with copper-sulphite minerals.

Therefore these alloys probably result from smelting copper ores containing arsenic or mixed copper-arsenic minerals, olivenite or clinoclase [5]. A golden colour characterises these alloys. Bronze, as an intentional alloy of copper and tin, began to be made between 3000–4000 b.C. probably with the intent to overcome the softness of “pure” copper (only marginally useful for the production of tools). The presence of tin increases both the alloy’s castability and the product’s hardness. When the tin content goes beyond 20% the alloy gets a silver-white and glossy appearance (mirrors were made by the Romans with an alloy called “speculum” holding a tin content of 19 - 33% and by the Chinese with a tin content ranging from 24 to 50%) [6-9]. The practice of adding limited amounts of lead (generally less than 2%) to bronze to increase workability and fluidity began around 1000 b.C. and was fully developed by the Romans. As lead segregates in the alloy (not soluble in copper) it can be recognised in a metallographic section as small dark spots. In what is called lead-bronze the lead content may be as high as 10% with the intent of improving the alloy’s softness and so castability (when fine details are of interest).

When the opposite result is desired, as for the production of cutting tools, which have to have a cutting-edge, antimony or arsenic can be added to bronze producing antimonial or arsenical bronze.

During the Empire of Augustus, the Romans began to add limited amounts of zinc to copper in order to improve castability and obtain a bronze-like alloy called brass [10].

Chinese smelters added zinc to bronze to improve workability and to obtain a whitish appearance. The presence of nickel (above 1%) was detected in bronze objects produced by the Sumerian and Syrian civilisations during 4000 – 3500 b.C. and in China, nickel was added to obtain a silverish appearance. Again antimony was found mainly in copper-base objects produced in tin-poor regions, like the Caucasus, [11].

The use of copper was and is probably so common and widespread^a^, not only due to its characteristic colour or its easy workability, but also thanks to its durability, as in former times an objects life-time was of high importance; the life-time of copper and copper alloy objects is high, when the right maintenance treatments are observed.

However, as a metal it is subjected to an oxidation phenomena and archaeological objects are always covered by more or less thick corrosion layers, depending on intrinsic (alloy composition and structural features) and extrinsic (e.g. soil characteristics) variables [12-14]. So, when the composition of an archaeological object is studied today, regardless of the method of analysis used, the fact that the measure obtained is not the exact representation of the original alloy composition [14] must be taken into consideration. Even if only the remaining metal bulk is analysed, its composition can have changed due to preferential corrosion of certain alloying elements and the leaching phenomena. Bearing this fact in mind, compositional studies can hardly aim to determine the exact concentration of an element [15] in the original ancient alloy but rather aim to detect intentional differences created by ancient metalworkers in order to answer the questions of archaeometallurgists and archaeologists.

**Figure 1:**
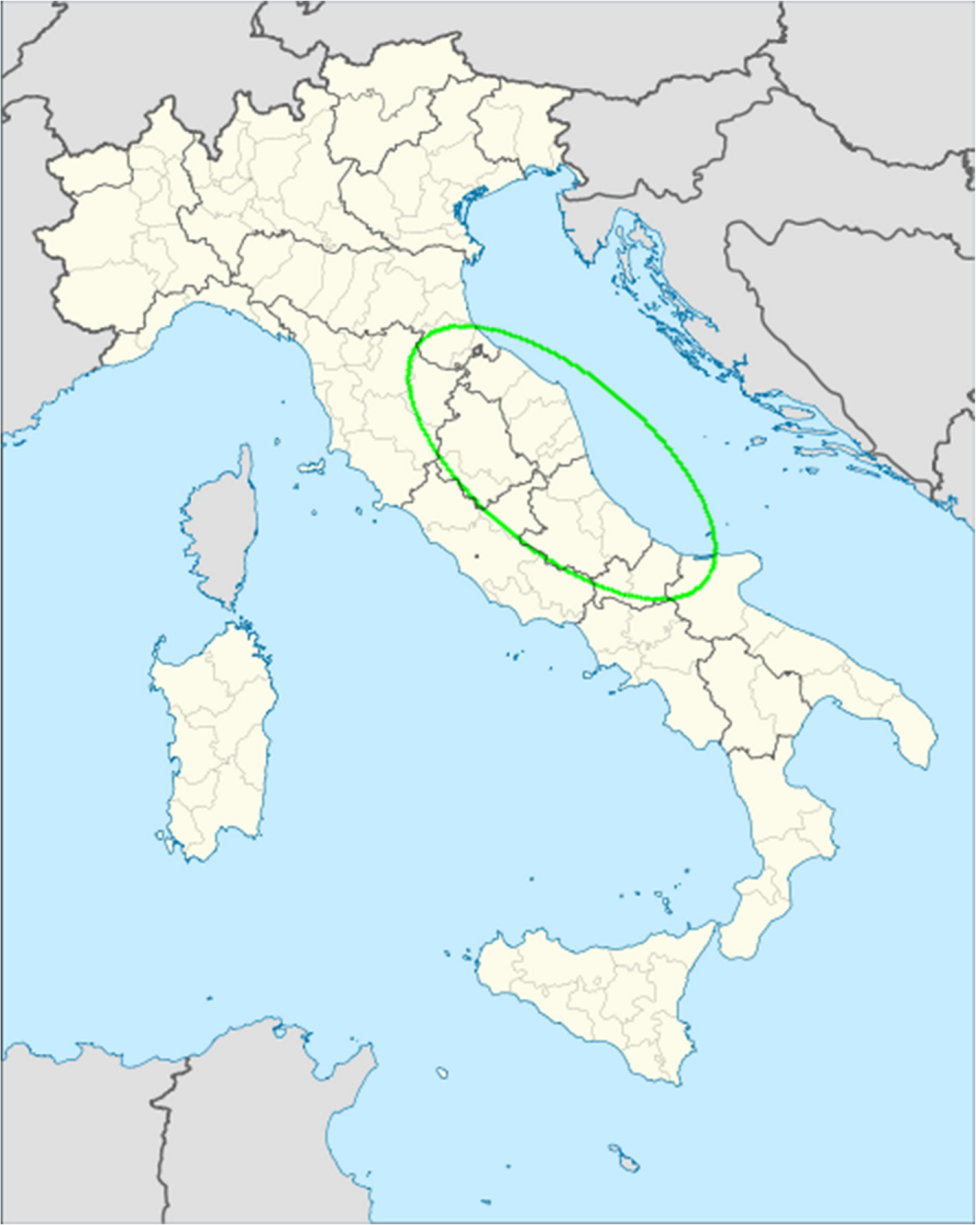
**Drawing of Central Italy; the circle signs the approximate provenience area of the studied bronze objects dating from Eneolithic to first Iron-Age.**

**Figure 2:**
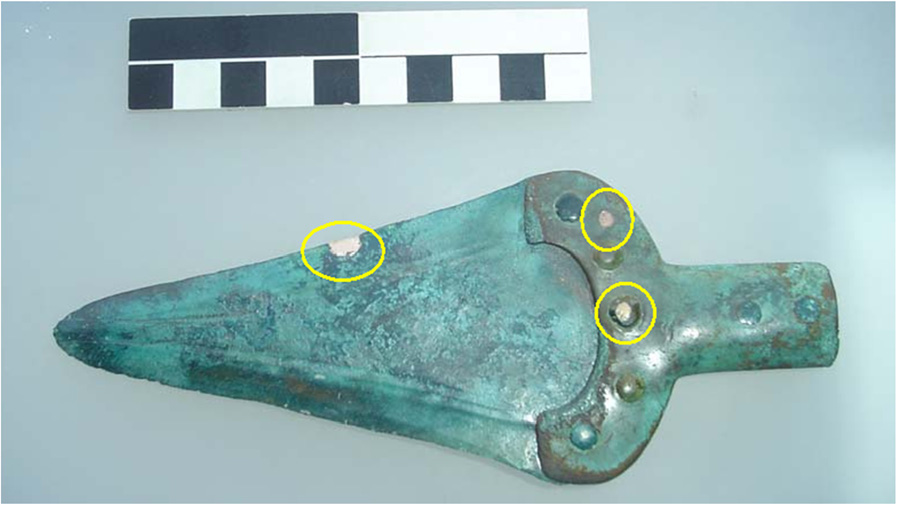
**Pugnale da Ripatransone: an example of patina abrasion for measurement- purpose can be seen; the abraded spots are enhanced by yellow circles.**

The aim of the present study was a new attempt [16] to detect a correlation between composition, age, type of object and its destination of usage; this time on a sample of 134 archaeological bronze objects excavated in Central Italy (mainly Abruzzo Region), see Figure 1, dating back from the Eneolithic Period to the end of Bronze Age including the early Iron Age. In Figure 2 a photo of a measured object is shown.

As in the previous work [16], compositional data was provided by micro-invasive ED-XRF analysis, but here object types were easily recognisable and thus so was their destination of usage; Table 1 summarizes data on the objects studied: usage (according to shape) and the number of measurements. Compared to other laboratory techniques for elemental analysis like AAS, IC or MS, [17] ED-XRF analysis is more widely used for bronze studies. This is mainly due to the instrument’s easy portability which, although unable to detect certain elements and lacking in accuracy, in any case allows class’ recognition based on main elements [18].

**Table 1:** **Daily use of the objects as recognised by archaeologists, number of measurements, number of sub groups**

**groups**		**n.measures**	**n.sub.groups**
groupsaxes, choppers, hatchets		42	9
poleaxes, spearheads		26	3
brooches and fragments		26	7
swords		23	8
knives, poignards		17	7
rings		12	3
razor		11	4
needles, pins		9	2
skull, ingots, metal foils		6	3
nail		6	1
iron damascene on bronze brooch		5	2
spring wire wound		5	1
sword sheath		4	1
arrowhead		4	2
sickle		2	1
earring		2	1
	total	200	55

To what extent did ancient foundries control the concentration of elements, and principally, were they able to/interested in recognising the composition of a broken object before reusing or re-melting it? These questions are the archaeological premise for the present study, centred not on the measurement methodology but aiming to investigate data correlation by particular chemometric treatment in order to confirm or reject the hypotheses that ancient metalworkers reused/recycled entire objects or parts of them.

## Results and discussion

Production zone, foundries in the central part of Italy and in particular the Abruzzo region no large quarries for mineral extraction for the production of bronze are present [19]; therefore bronze was obtained either by metallurgical processes with expensive imported minerals or probably, by recycling bronze objects. The compositional heterogeneity of Abruzzan bronze antiquities, also highlighted in this work, suggests that production was very often based on recycling processes.

The most antique items in this study are dated back to the Eneolithic Period; among them is a small axe of almost pure copper, probably one of the few objects made with rare native copper.

The majority of the antique bronze items considered in this study are objects of the “ripostiglio di Alanno”.

Archaeometric investigation brought to light that in this period objects were already made with a tin rich bronze alloy, with tin content even higher than 10%.

Also the more recent pieces, either of the Bronze or Iron Age, show a relatively high tin content; this is probably related to the improvement of the mechanical resistance of such an alloy. Furthermore, tin can accumulate in the corrosion patina, as observed on an object found near Sulmona, where the tin content reaches up to 15%.

### Sampling method

Any Exploratory Data Analysis (EDA) starts with sampling; of course this chemometric analysis starts from a collection of objects stored in various museums in central Italy (measurements were conducted under the auspices of a CNR project started in 1997 with the aim to ameliorate the scientific knowledge on the immense inheritance of objects). After studying the variety (type and age) of the conserved objects, we selected a probability proportionate to size sampling method. A data set should contain a certain level of redundancy to ensure the method of calculation’s precision (and/or homogeneity of alloy); in this case a third of the objects were measured twice or more.

### Elemental analysis

For each object at least one measurement point was identified; selection criteria for the measurement point was finding a way to remove the patina without damaging the object. An example of patina removal to access to the “original material” is shown in Figure 2. and in Fig. A (please note that only figures named with numbers are shown in the text, while those named with Capital letters are located in the (Additional file 1)). The patina was abraded with a tiny diamond sphere to avoid contamination by any other metal and three ED-XRF measurements were performed (see Fig. B in the Additional file 1); their mean was then considered as “bulk” composition. When possible, an object was analysed in 2, or up to 5 points; in this case the collected data should allow estimation of the repeatability of the system object/instrument taking into account the heterogeneity of the antique alloy. Two different types of repetition were performed; 3 times on the same point without moving anything to evaluate the “precision” of the method and then on different points of the object to evaluate the “homogeneity” of the alloy.

The next step was the computation of the statistic average value of detected elements, shown in Table 2. Fortunately, the average value of the 134 objects is in accordance with the typical composition of Bronze objects for the middle and late Bronze Age in Italy and France [9,20].

**Table 2:** **General statistic values from 200 measurements on the 132 bronze objects**

**general statistic, all**	**Cu%**	**Sn%**	**Pb%**	**Fe%**	**Zn%**	**Sb%**	**Ag%**	**As%**
smallest value	45.50	0	0	0	0	0	0	0
largest value	99.90	17.70	7.60	49.90	9.20	0.80	0.70	0.60
average (arithmetric mean)	88.32	8.80	1.60	0.93	0.13	0.12	0.10	0.01
sample population variance	38.17	12.33	2.14	26.99	0.56	0.03	0.01	0
sample standard deviation	6.18	3.51	1.46	5.20	0.75	0.18	0.12	0.07
harmonic mean	87.71	n.a.	n.a.	n.a.	n.a.	n.a.	n.a.	n.a.
geometric mean	88.05	n.a.	n.a.	n.a.	n.a.	n.a.	n.a.	n.a.
most frequently occurring value	91.20	7.30	0	0	0	0	0	0
sample kurtosis	19.43	−0.21	3.06	75.50	115.96	5.61	4.27	48.42
sample skewness	−3.15	0.02	1.57	8.48	10.26	2.34	1.77	6.90
standard error of the sample mean	0.44	0.25	0.10	0.37	0.05	0.01	0.01	0
95% magnitude of the confidence interval	0.86	0.49	0.20	0.72	0.10	0.03	0.02	0.01
diff% avg/mediane	−0.89	1.73	18.67	100.00	100.00	19.35	−2.56	100.00
median value in list	89.10	8.65	1.3	0	0	0.1	0.1	0
sample percentile, value at 5%	80.40	3.30	0	0	0	0	0	0
sample percentile, value at 95%	95.81	14.81	4.51	1.20	0.60	0.60	0.30	0
90 percentile difference (see two above cells)	15.41	11.51	4.51	1.20	0.6	0.60	0.30	0
interquartile difference (3^-1^)	5.33	4.83	1.70	0.40	0	0.20	0.10	0
outliers ? (using lower limit = avg- 3×std.dev)	69.78	−1.73	−2.79	14.66	−212	−0.42	−0.26	−0.20
outliers ? (using lower limit = avg + 3×std.dev)	106.85	19.34	5.98	16.52	2.37	0.67	0.46	0.22
outliers ? (2.5% percentile)	78.57	2.49	0	0	0	0	0	0
outliers ? (95.5% percentile)	96.72	15.4	5.61	2.03	0.9	0.8	0.4	0
NUM. OF ZEROS	0	3	25	105	177	87	87	195
counts measures (n. objects)	200	200	200	200	200	200	200	200

In the field of multivariate analysis and chemometrics the word “classification” has a well known meaning. Therefore, in the present paper we tried to avoid this term and to substitute it with the term “group”, because we operated a categorization of objects which was formerly labeled with standard criteria and then differentiated them using an Attribute-Value system. After some discussion, we decided to use 3 criteria: age, shape and daily usage. After suggestions from an expert restorer, another criterion was added and thus another subdivision obtained. Further discussion produced further grouping methods; therefore 5 different Attribute-Value systems were produced, shown in Table 3 (with group dimension and selection criteria).

**Table 3:** number of bronze objects in each group using the Attribute-Value classification method (4 researchers and daily use), * not used in the PCA analysis

**builder**	**gr.name**	**how many obj.**	**builder**	**gr.name**	**how many obj.**
GGroup	1, eneolithic	2	USEgroup	1, sword	10
	2, antique bronze	21		2, needle	9
	3, middle bronze	14		3, spearhead	19
	4,middle-late bronze	7		4, rings	9
	5, late bronze	33		5, axe	27
	6, final bronze	22		6, ingots	6
	7, final bronze, 1^ Fe	19		7, nail	4
	8, 1^Fe	11		8, knife	10
*	9, VII-VI a.C.	5		9, sickle	1
GVgroup	1, ornaments	32		10, brooch	17
	2, sharpened	15		11, sheath	2
	3, axes	27		12, earring	1
	4, swords	12		13, arrowhead	4
	5, spearheads	18		14, razor	5
	6, ingots	6		15, spring wire	5
	7, sheaths	2	*	16, damascene Fe rich	5
	8, pointed, heavy	8	RRgroup	1, flat blades	28
	9, pointed, light	9		2, pointed, tip	14
*	10, damascene Fe rich	5		3, long blades	26
SHPgroup	1, pointed	59		4, cutting blades, knives	12
	2, sharp	64		5, wire or wirewound	41
	3, for foundry industry	6		6, fodero di spada	2
*	4, Fe rich objects	5		7, for foundry industry	6
			*	8, Fe rich objects	5

Measurement repeatability should be evaluated; however, when different compositional values were registered on different spots on the same object, deciding whether they were due to the alloy’s very plausible heterogeneity or if there was a repeatability problem, was not possible. This is a common problem when working on archaeological material. However, initial calibration measurements also undertaken for quantification purposes on material with certified and similar composition should guarantee accuracy. 44 objects were measured twice or more; a moon shaped razor was measured seven times and obtained values are given as an example in Table 4, which shows a possible method for differentiating the reproducibility of the system instrument/object from the alloy heterogeneity. The measurements 838–1, 838–2, 838–3 were performed on the same point and the measurements 838–21, 838–22 on a different point; also 838–31 and 838–32 were performed on yet another point. Although the razor was a special case, it was measured in 3 different points, so obtaining 3 + 2 + 2 measurements.

**Table 4:** **The razor with moon shape measured in 3 points by ED-XRF, obtained values and Median Absolute Deviation from median for the point 1, repetition, and other points**

**note**	**code**	**Cu%**	**Sn%**	**Pb%**	**Fe%**	**Zn%**	**Sb%**	**Ag%**	**As%**
point n.1	838-1	86.6	9.0	3.8	0	0	0.3	0.4	0
point n.1	838-2	85.8	8.6	5.2	0	0	0.2	0.2	0
point n.1	838-3	86.8	7.4	4.5	0	0	0.7	0.6	0
point n.2	838-21	85.0	8.9	5.6	0	0	0.3	0.3	0
point n.2	838-22	87.4	6.6	4.8	0	0	0.7	0.5	0
point n.3	838-31	85.4	10.2	3.7	0	0	0.3	0.4	0
point n.3	838-32	80.4	13.1	6.3	0	0	0.1	0.1	0
point n.1	median	86.6	8.6	4.5	0	0	0.3	0.4	0
	arith. mean	86.4	8.3	4.5	0	0	0.4	0.4	0
	std.dev.	0.5	0.8	0.7	0	0	0.3	0.2	0
	MAD	0.2	0.4	0.7	0	0	0.1	0.2	0
n1, n2, n3	median	85.8	8.9	4.8	0	0	0.3	0.4	0
	arith. mean	85.3	9.1	4.8	0	0	0.4	0.4	0
	std.dev.	2.3	2.1	0.9	0	0	0.2	0.2	0
	MAD	0.8	1.3	0.8	0	0	0.1	0.1	0

The arithmetic mean, the median, the standard deviation and the median absolute deviation from median were calculated as well.

Table 5 shows the spread among measures obtained on the 4 objects with measurement repetition (3 times on the same point (see Table 6), and the 7 objects measured on more than 3 points (see Table 5) using the Median Absolute Deviation as indicator.

**Table 5:** **Median Absolute Deviation from median, same object measured in some points**

**n, code**	**visual classification**	**n. points**	**Cu%**	**Sn%**	**Pb%**	**Fe%**	**Zn%**	**Sb%**	**Ag%**	**As%**
60895-5	Ascia a margini rialzati	5	1.80	1.10	0.30	0.00	0.30	0.00	0.00	0.00
106647-4	Spada tipo Allerona	4	0.50	0.40	0.05	0.00	0.00	0.00	0.00	0.10
156421-4	Fibula con arco a gomito	4	0.25	0.35	0.05	0.25	0.00	0.00	0.00	0.00
41-4	Fibula con arco a gomito	4	0.40	0.25	0.45	0.25	0.00	0.05	0.05	0.00
35889-4	Anello con anellini	4	1.95	1.85	0.10	0.00	0.00	0.05	0.00	0.00
23923-4	Spada ad antenne tipo Fermo	4	0.55	0.60	0.10	0.00	0.10	0.05	0.00	0.00
24468-4	Fibula con arco ad serpeggiante a	4	3.50	1.75	0.55	0.20	2.40	0.05	0.05	0.00

**Table 6:** **Median Absolute Deviation from median, the 3 repetitions on the same point**

**n, code**	**visual classification**	**n. repeat**	**Cu%**	**Sn%**	**Pb%**	**Fe%**	**Zn%**	**Sb%**	**Ag%**	**As%**
67516-3	Spada tipo Allerona	3	0.20	0.00	0.00	0.00	0.00	0.00	0.00	0.00
67-3	Spada tipo Allerona	3	1.20	0.30	0.20	0.10	0.00	0.00	0.00	0.10
362-3	Fibula con arco a gomito	3	1.90	0.50	0.10	0.00	0.00	0.00	0.00	0.00
838-3	Fibula con arco a gomito	3	0.20	0.40	0.70	0.00	0.00	0.10	0.20	0.00
median of MAD for the 4 objects, same point			0.70	0.35	0.15	0.00	0.00	0.05	0.00	0.00
median of MAD for the 11 objects			0.6	0.4	0.1	0.0	0.0	0.1	0.0	0

Obtained statistical values are in accordance with typical data produced by applying an ED-XRF method.

Before putting the measurements obtained on different points of the same object together, using a central value descriptor, one must check for outliers. In Table 7 the 200 measurements were evaluated to find possible outliers; therefore values beyond the 95 percentile (median centred) are shown in bold.

**Table 7:** **Outliers analysis, in bold the values out of 2.5 percentile on the 2 sides**

**timeline**	**n.code**	**visual classification**	**Cu%**	**Sn%**	**Pb%**	**Fe%**	**Zn%**	**Sb%**	**Ag%**	**As%**
eneolithic	41	Axe, broaded borders	**99.9**	**0**	0	0	0	0	0.1	0
eneolithic	60898-1	Axe, flat shape	**97.9**	**0.3**	0.4	0.4	0.9	0.1	0.1	0
eneolithic	60898-2	Axe, flat shape	**99.5**	**0**	0	0.3	0	0.1	0.1	0
middle bronze	36521	Sword, Pertosa type	85.8	13.3	0.2	0.2	0	0.1	0	**0.4**
middle to late bronze	27914	Arrowhead, with spigot	82.8	**15.6**	1.5	0	0	0	0.1	0
middle to late bronze	106647-1	Sword, Allerona type	86	12.8	0.6	0	0	0	0.1	**0.6**
middle to late bronze	106647-1	Sword, Allerona type	87	12	0.5	0	0	0	0	**0.4**
middle to late bronze	106647-1	Sword, Allerona type	86.5	12.4	0.6	0	0	0	0	**0.5**
middle to late bronze	106647-1	Sword, Allerona type	87.9	11.5	0.4	0	0	0	0	**0.2**
late bronze	67-3	Knife Celano type	82.3	**15.5**	1.4	0.6	0	0	0.1	0
late bronze	31212	Brooch with eyelet	**97.3**	2.7	0	0	0	0	0	0
late bronze	6	Brooch with eyelet	96.7	**2.1**	0.8	0	0	0.1	0.2	0
final bronze	35780	Skull, casting	**99.8**	**0**	0	0.2	0	0	0	0
final bronze	31665	Small rod	89.8	7	0.8	0.8	0	0.8	**0.7**	0
final bronze, 1^ Fe	14228	Leaf shape spearhead	76.3	14.8	**7.6**	0	0	0.8	0.4	0
final bronze, 1^ Fe	14226-2	Spearhead	83	10.4	**5.9**	0.5	0	0.1	0.1	0
final bronze, 1^ Fe	14224	Spearhead	79.8	12	**7.2**	0.3	0.8	0.6	0.2	0
final bronze, 1^ Fe	14218-1	Spearhead	81.8	10	**7**	0.2	0	0.8	0.2	0
1^ Fe	23924-2	Sword sheath, Guardiavomano type	80.4	**16.8**	2.6	0	0	0.1	0.1	0
1^ Fe	838-3	Razor, Vulci type	86.8	7.4	4.5	0	0	0.7	**0.6**	0
1^ Fe	838-22	Razor, Vulci type	87.4	6.6	4.8	0	0	0.7	**0.5**	0
1^ Fe	838-32	Razor, Vulci type	80.4	13.1	**6.3**	0	0	0.1	0.1	0
1^Fe	24468-1	Brooch, arc and snake	**69.7**	**17.7**	2.2	1.2	**9.2**	0	0	0
1^Fe	24468-2	Brooch, arc and snake	**77.5**	14.1	3.3	0	**4.8**	0.1	0.1	0
1^Fe	24468-4	Brooch, arc and snake	78.7	**16.2**	4.8	0	0	0.2	0	0
VII-VI a.C.	28871	Iron damascene on bronze wire	78.6	3.1	0	**18.3**	0	0	0	0
VII-VI a.C.	17101	Iron damascene on bronze brooch	**48.1**	3.3	0	**48.6**	0	0	0	0
VII-VI a.C.	17097	Iron damascene on bronze brooch	**45.5**	4.5	0	**49.9**	0	0	0	0
V-VI a.C.	25689	Iron damascene on bronze wire	81.1	7.3	0.5	**11**	0	0	0	0
V-VI a.C.	25689	Iron damascene on bronze wire	78.6	6.7	0.4	**14.3**	0	0	0	0
		outliers ? (2.5% percentile)	78.57	2.49	0	0	0	0	0	0
		outliers ? (97.5% percentile)	96.72	15.40	5.61	2.03	0.90	0.80	0.40	0.005

The first decision criterion in the outlier analysis was to maintain all objects with only one outlier element, reducing the table by half. Immediately, the 5 measurements on the 5 Agemine containing both Fe and Cu were enhanced. All grouping attempts place these 5 objects in a separate group and thus they may be excluded for further computation. Further analysis of the table enhanced three objects of the very early Bronze Age, made from almost pure copper; being the only objects of this period, they could not be excluded. Then there is object n° 35780, a casting residue made from almost pure metal; this object is, not an outlier. Lastly, object n° 14228 could be an outlier due to high Pb, but careful table and raw data reading showed that all spearheads have an important Pb and Sn % (even if they come from the same settlement) and thus it will be kept, as well.

As a result, outlier analysis EDA has proved helpful, enhancing groups with extreme composition.

Unfortunately, the Dixon and/or Grubbs outlier test is not applicable because our distribution is not normal and the number of objects is too large (maximum number is 30 to 100 [21]).

At this point it becomes clear that, instead of using other non parametric methods like Peirce [22,23] or Chauvenet [24], a debatable method was used: objects were checked beyond the 95th percentile centred on the median; the same idea is sustained by MAD in Table 5.

In the electronic Additional file 1, frequency distribution charts of single elements were included : Fig. C-J.

After removing the outliers, the resultant matrix had dimensions of 129 rows by 7 columns (objects/metals): this is because 5 Fe-rich objects (falsely assigned to be bronze) were omitted. Furthermore, the metal As, detected in only 2 out of 134 objects from the matrix, was removed because it was identified as an outlier during the PCA analysis of the transpose matrix, where elements are treated as objects.

### Data analysis

Matrix scaling often has a distorting or simplifying effect on successive multivariate data treatment [25].

To enhance the influence of scaling on data, Box-Whiskers plots are contained in the Additional file 1: Fig. K shows raw data, Fig. L shows column-centring, Fig. M autoscaling and Fig. N log-ratio scaling. Based on experience treating datasets consisting of around 100 objects using the ED-XRF technique and following recommendations [26-28] log-scaling was selected for column pre-treatment.

Compositional data deriving from WD or ED-XRF measurements often contain a high number of “zero” values for the minor alloy elements; classification based on this data is a problematic issue. “In compositional data analysis we distinguish two kinds of zeros: essential zeros - or absolute absence of the part in the observation - and rounded zeros – or presence of a component, but below detection limit” [29]. Distinction of the two types of zeros is not possible if values are closed to 100% on the object row, as in the present case.

**Figure 3:**
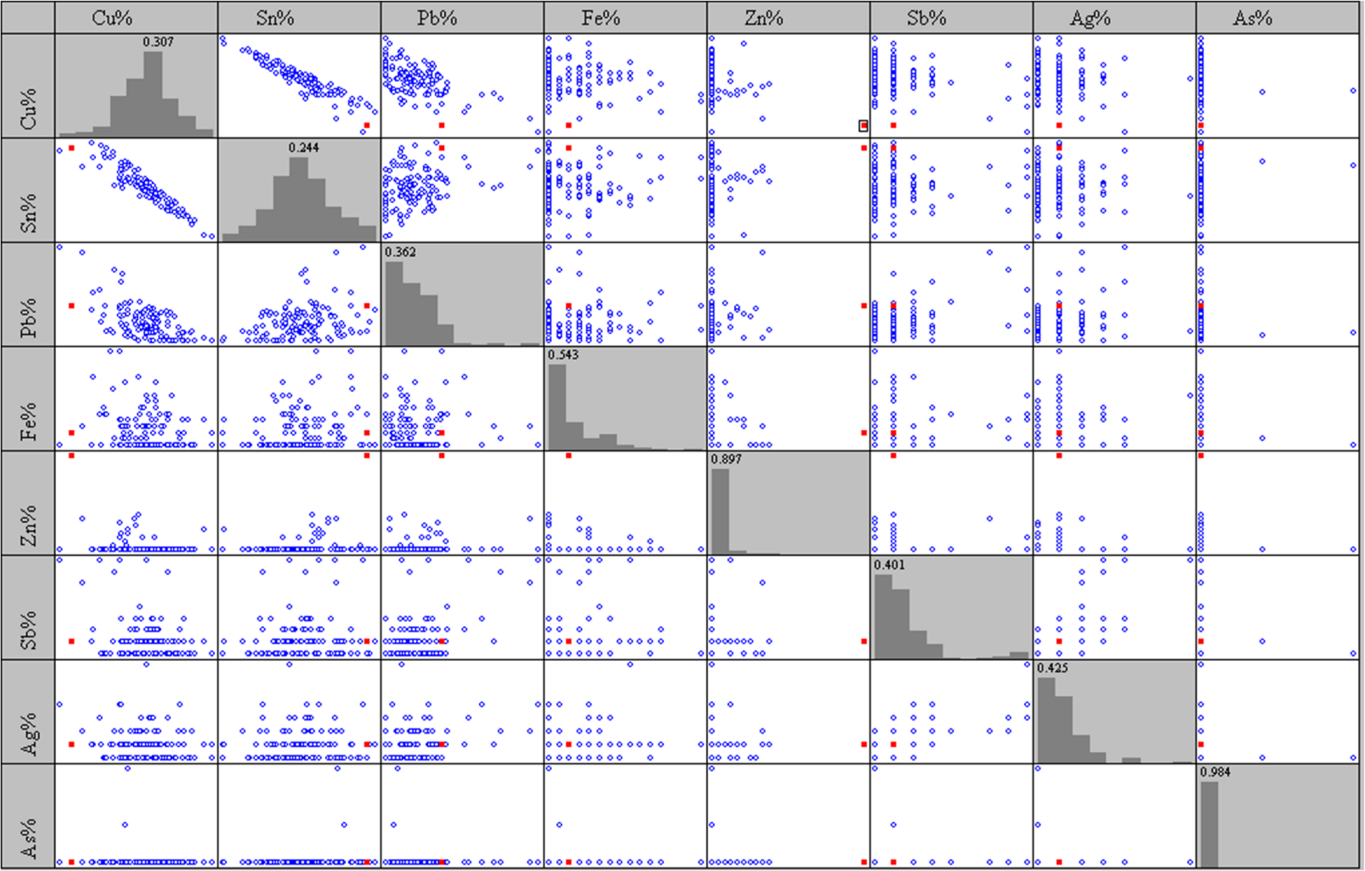
**Scatter Plot Matrix (SPLOM) for the 8 elements and 129 objects with the aim to identify correlation among data and doubtful values.**

A first attempt to separate groups was obtained using a scatter plot-matrix, without matrix pre-treatment, showing all possible var-var combinations. The scatter plot matrix graph (SPLOM) in Figure 3 shows that bronze objects are concerned as only the Cu-Sn scatterplot shows correlation. One object with high Zn is highlighted in red. This EDA graph method was useful for detecting particular situations like that of object n. 24486 (a fibulae measured in 4 points, see the Additional file 1): we can see the low value of Cu, the high value of Sn, but remaining elements show central values with respect to their distributions.

**Figure 4:**
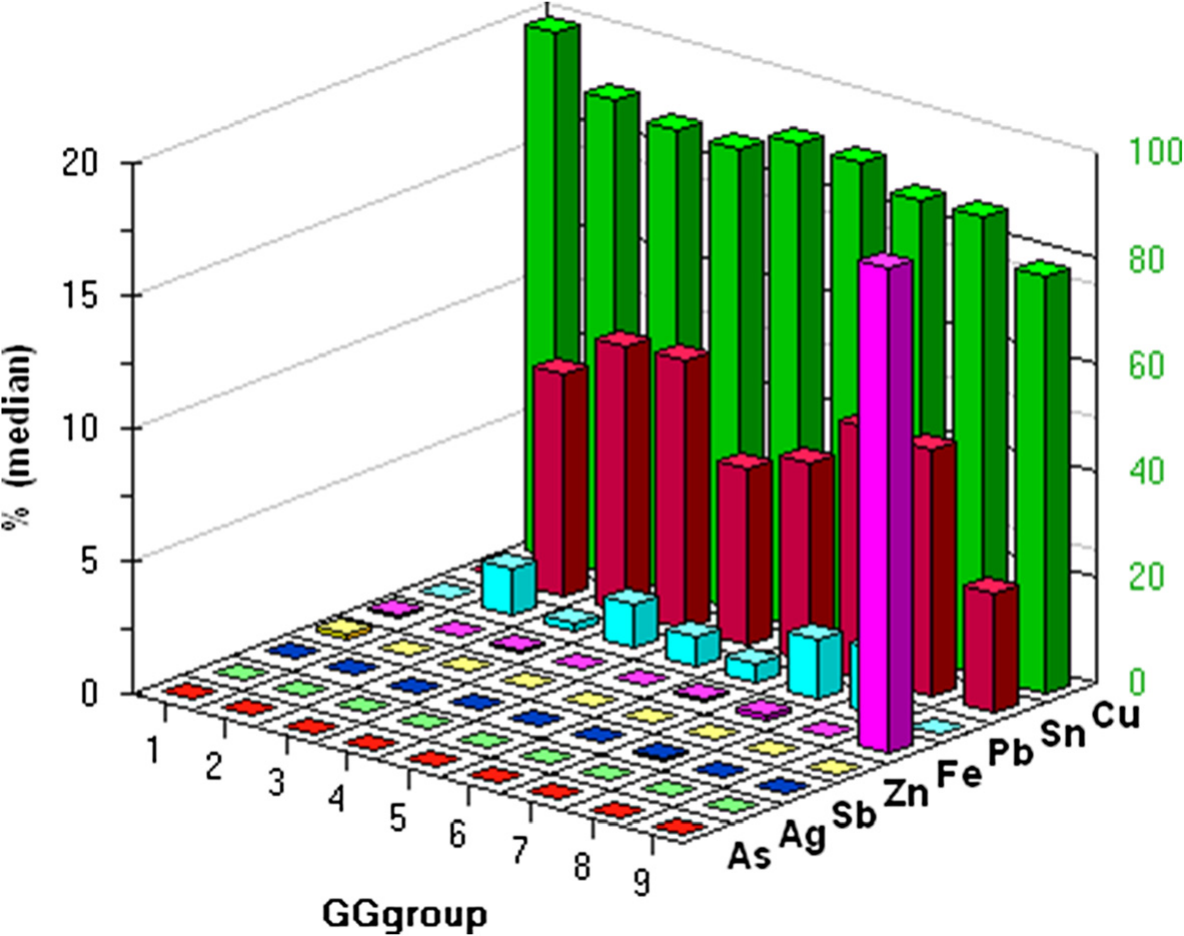
**3D-histogram of groups based on objects’ age (by author GG).** There is a tendency for Cu reduction, high variance for Sn and not casual presence of Pb. The green scale on the right refers to Cu, while the black scale on the left refers to all other elements.

A 3D histogram with median values for each element was used in an attempt to identify whether there was a correlation between composition and grouping (5 groups as decided by the authors). Fig. O to S (Additional file 1) show all obtained histograms. No solution to our problem seems evident. Only Fig. P, corresponding to Figure 4 in this paper, shows a decrease of Cu with age and a consequent increase of Sn in two steps, continuous variation of Pb, which is certainly intentional.

In the Additional file 1, the median of the concentrations of elements for all 134 objects is presented; various graphs, including the High Low Open Close (HLOC) for each metal and group, are shown. Again none of these graphic representations seem to indicate a solution to our problem. In similar situations, multivariate analysis has often offered a satisfying solution, as in the case of identifying different types of Imperial age marble [30], and the individuation of characteristic parameters of “glass paste” [31], where an unsupervised technique like Principal Component Analysis (PCA) enabled the identification of object groups which were not detectable by former mathematic and graphic methods.

**Figure 5:**
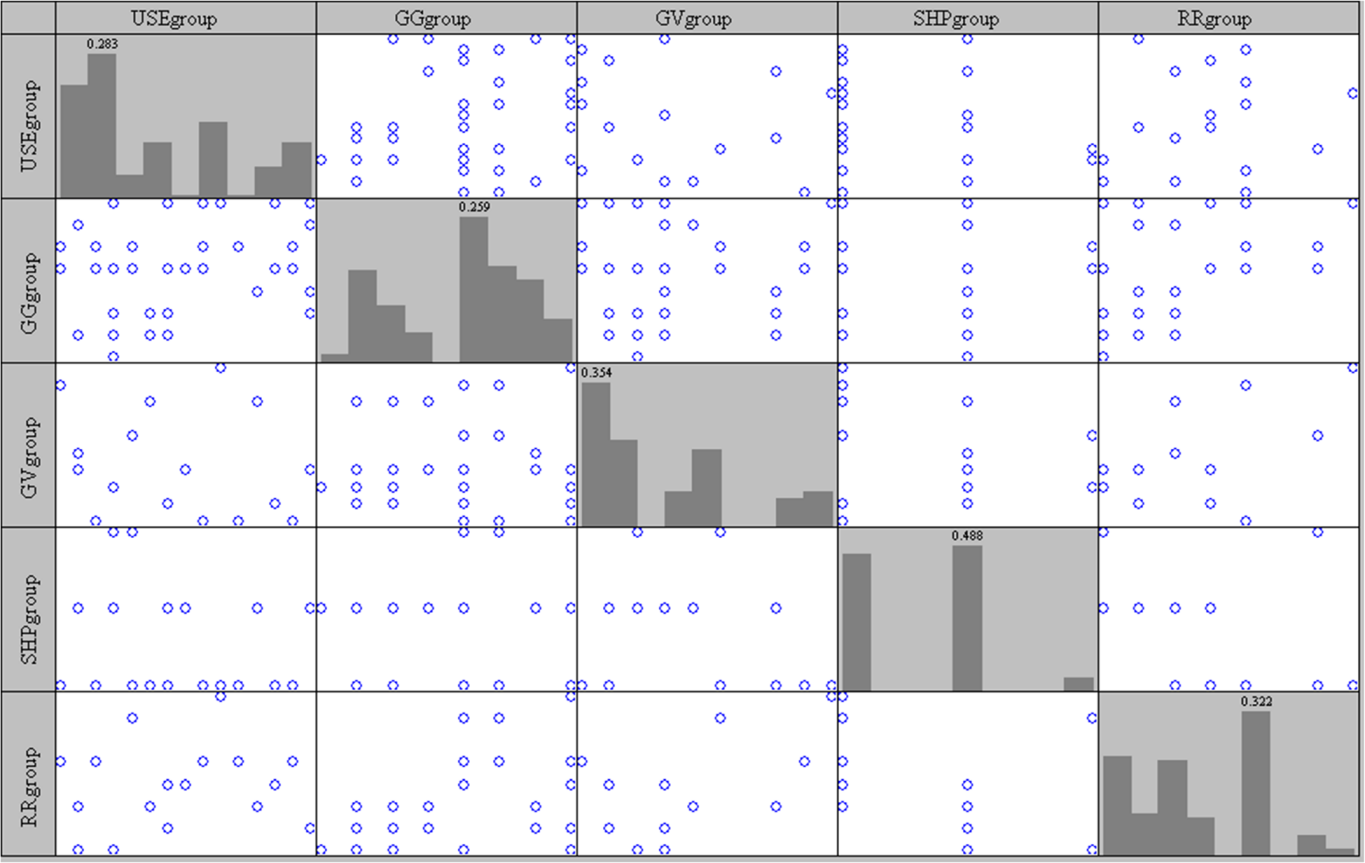
**SPLOM of all Attribute-Value classification groups to check for correlation and to visualize the different ways of grouping objects.**

The scatterplot matrix in Figure 5 (and Fig. T) is interesting; the Attribute-Values groups, as decided by the authors, are not correlated and thus it is reasonable to repeat projections for all group scores.

**Figure 6:**
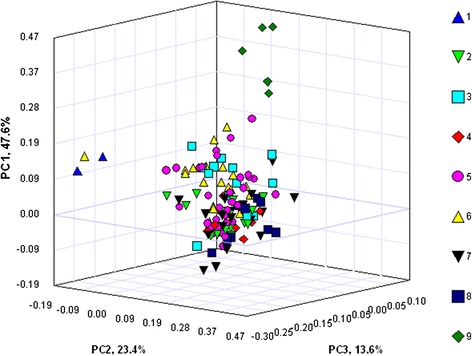
**PCA scores chart for all 134 objects and after log-ratio and column centring pre-treatment; using the GV grouping method described in Table** 3 **only 2 groups are enhanced: one with the Fe rich objects (upside) and one with the Eneolithic objects (on the left).**

Chemometric analysis was performed in an attempt to visualise clouds of objects through PCA using all elements and also including the Agemina group. In Figure 6 the 3D representation of all objects and “usage groups” is shown. Weak separation is only evident in one group, on the upper right, composed by Fe-rich objects. This finding is encouraging for the development of the model and successive results.

Another small group on the far left of the chart can be identified, Although recalculation without the Fe-objects would probably produce better separation.

**Figure 7:**
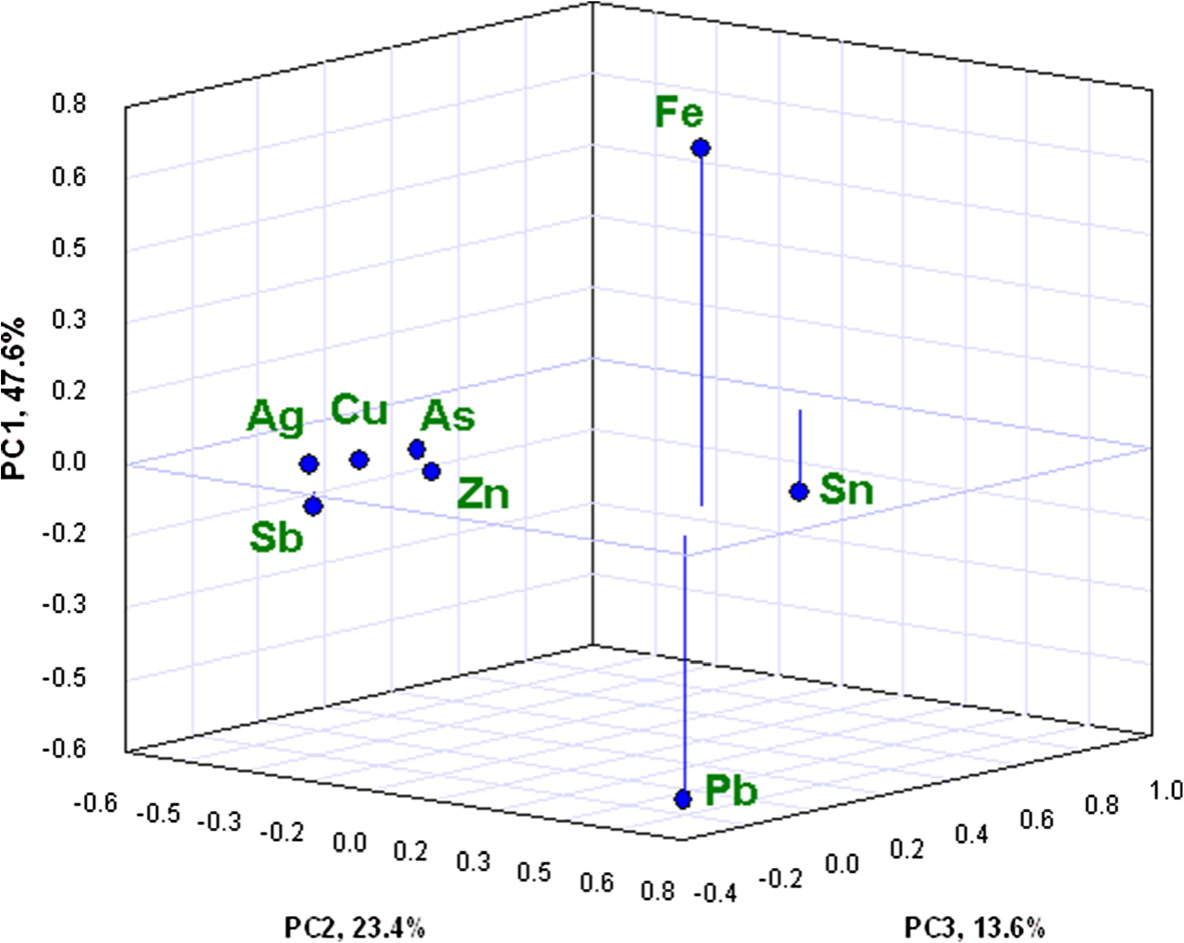
**PCA loadings chart for all 134 objects and all elements, after log-ratio and column centring pretreatment.** The contribution of Cu is almost null for PC1 and very small for PC2 and PC3.

Figure 7 shows the loadings for all 134 objects and the 8 detected metals.

**Figure 8:**
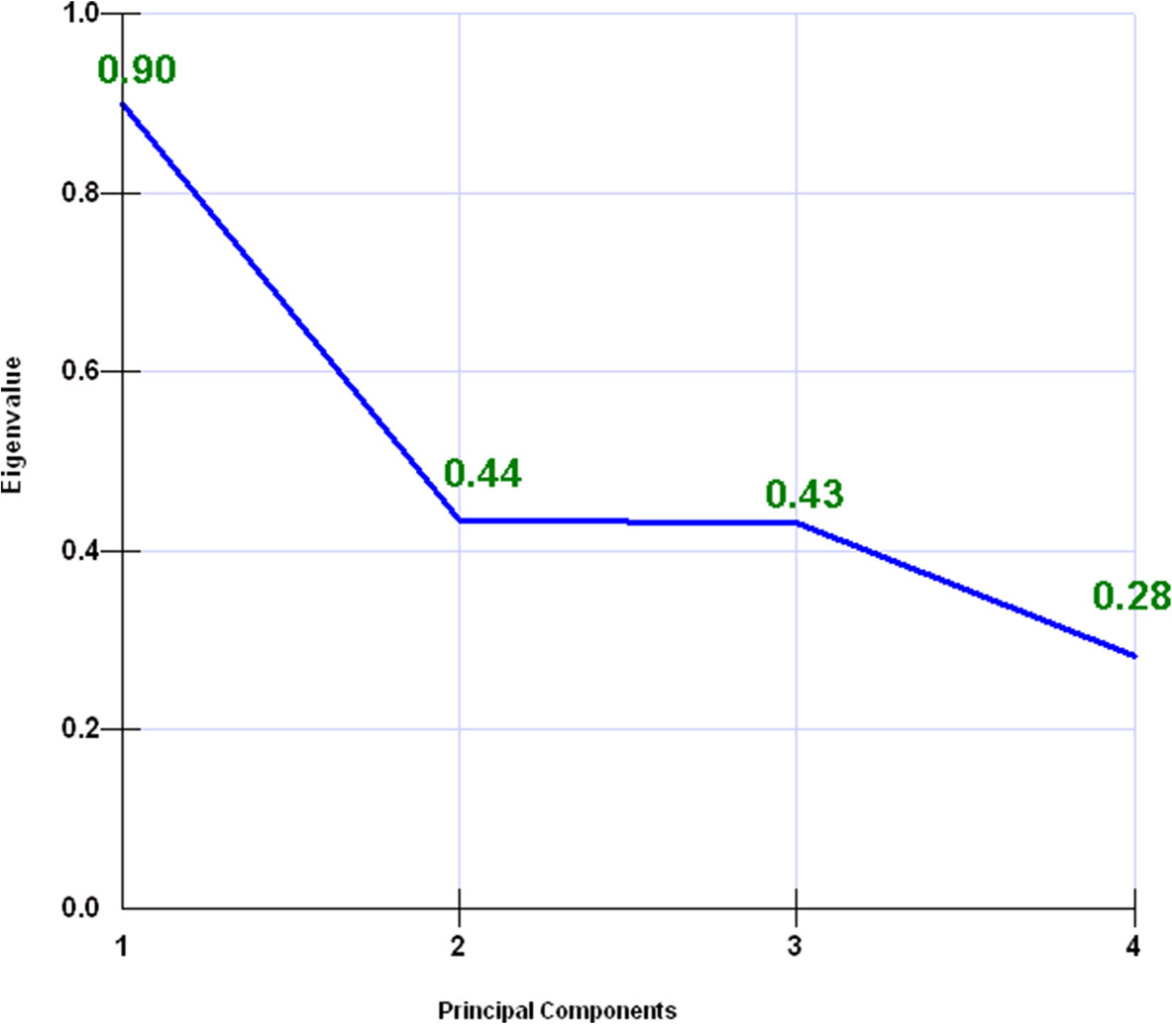
**The PCA scree-plot for the finally considered 129 objects (As left out).**

**Figure 9:**
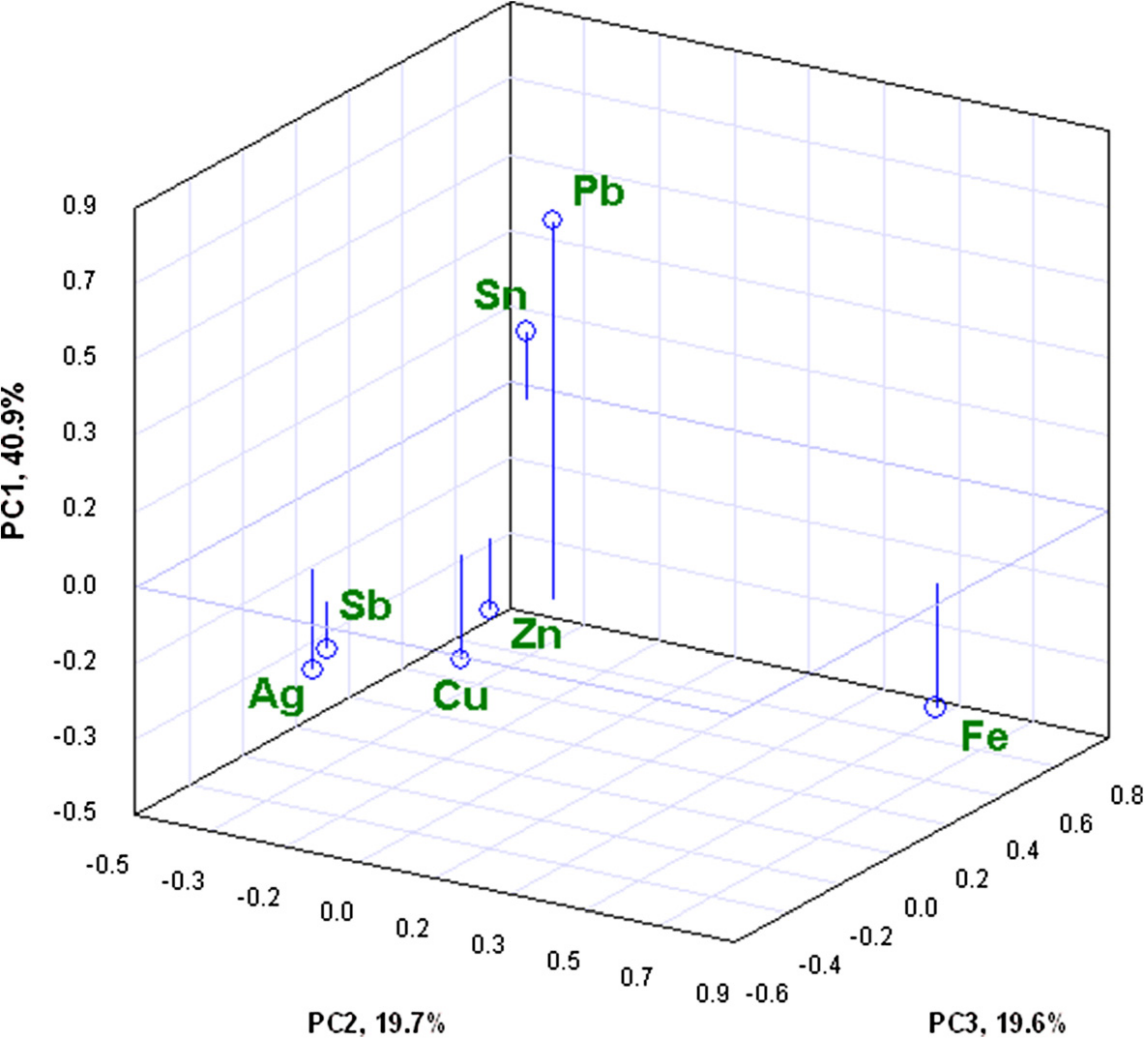
**3D representation of the PCA loadings for the finally considered 129 objects (As left out); contrarily to Figure** **9** **all elements contribute significantly to the projection of the objects in the score space.**

Based on these representations, we decided to exclude the 5 Fe-rich objects, which compressed the potential separation of other objects, and to exclude As because, as shown in Table 2, it was detected in only 5 out of 200 measurements (e.g. in objects n° 36521- a nail - and n°106647 - a sword). With these exclusions we obtained the previously cited matrix dimensions (129 × 7) with log-ratio centring as matrix pre-treatment; the ScreePlot in Figure 8 suggests visualisation of the first three PCs. In Figure 9 the new loadings are shown confirming that the 3rd component has an indisputable importance. Figure 9 shows high values of Pb (positive) and Fe (negative) for PC1; only Fe has a positive high value in PC2, while Zn and Sb are in opposition in PC3.

**Figure 10:**
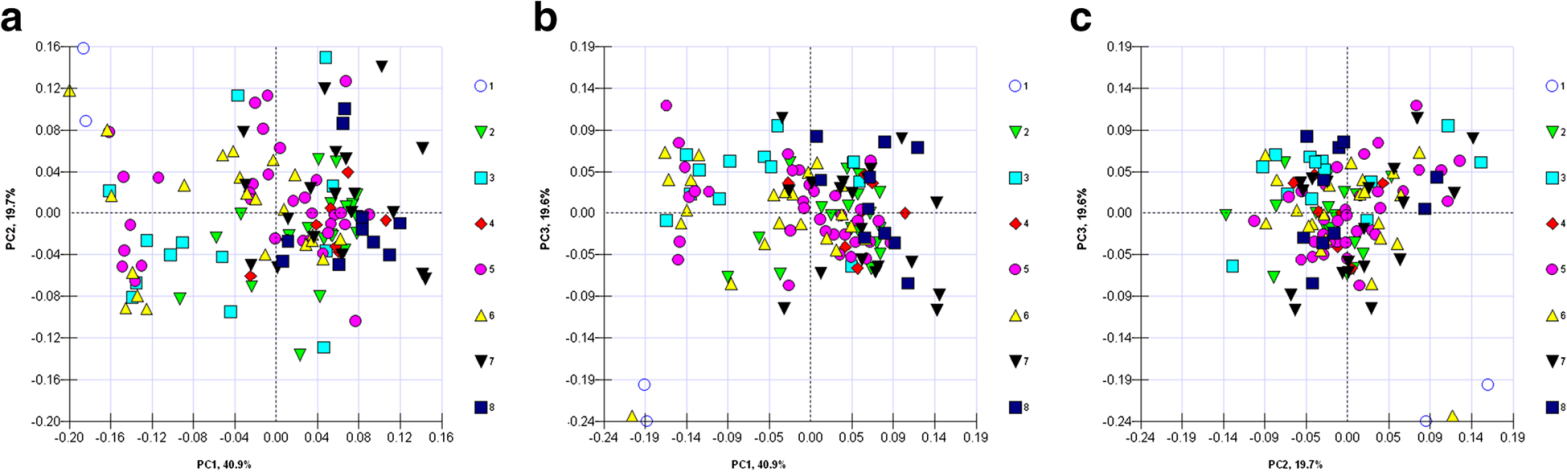
**PCA analysis, score plots of 129 objects, 7 elements.** Colour markers reflect object grouping by the age criterion (author G.G). **a** PC1 vs PC2 scores; **b** PC1 vs PC3 scores, Cu rich objects are grouped in the bottom left corner; **c** PC2 vs PC3 scores with Cu rich objects in bottom right. Using the same scale as in Figure 11 the constriction of the point cloud becomes evident.

In Figure 10a-c the scatterplot of the scores are shown for different combinations of the first three PCs, using the Attribute-Value grouping method suggested by author G.G. (based on supposed age of production).

**Figure 11:**
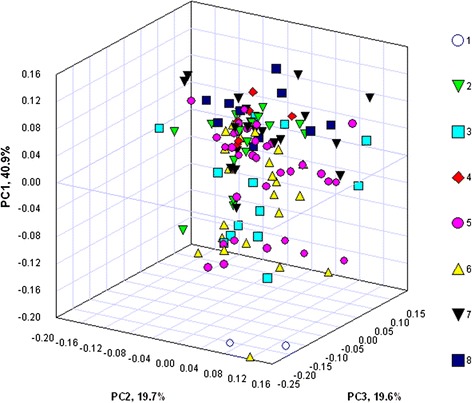
**PCA scores in 3D representation (129 objects, 7 elements).** Colour markers reflect object grouping by the age criterion (author G.G), so the chart is the combined 3D representation of Figure 10a, b and c.

In Figure 11 the three previous graphs are shown from a different point of view (3D) for better visualisation of the objects’ projection; colours refer to object grouping as suggested by author GG.

In the Additional file 1 we included representations of PC vs. PC in the plane and in 3D for all 5 grouping methods; for example, PC1 vs PC2 in Fig. V, PC1 vs PC3 in Fig.W, PC2 vs PC3 in Fig. X and the 3 axis in Fig. Y for the USE group. Similar ones are shown from Fig. Z to Fig. AP; the filenames should be explicative for content description.

PCA is unsupervised, it is not a classification method and the computation does not take into account our 5 defined grouping methods, which were thus visualized simply using different coloured markers; Table 8 shows the eigenvalues, RSS and PRESS values. Table A in the Additional file 1 shows the matrix after pre-treatment and the scores and loadings used for all following charts.

**Table 8:** **Principal Component Analysis, crossvalidation, eigenvalues and related percentages (after log-ratio, −As), using MVA add-in for Excel by R.G. Brereton**

**N. PCs**	**RSS**	**PRESS**	**E-value**	**%**
#1	165.930	175.847	0.914	41.346
#2	109.555	148.885	0.440	19.927
#3	55.506	68.195	0.422	19.105
#4	20.040	23.488	0.277	12.538
#5	6.479	7.981	0.106	4.793
#6	1.64E-05	n/a	0.051	2.291
Total SS	5017.054	5017.054		

According to our customary approach to open discussion and sharing of data and results, in the Additional file 1 we included an .xls file with the raw data of all 134 measurements, the median and for the final 129 objects considered, we included principal statistics, correlation and covariance matrixes, a series of sheets with different grouping proposals and some further graphs; in addition, log-ratio values for further elaboration can be found.

### Experimental

… *no analysis is better than the sample itself*…[32], so extreme care is required for object selection, instrument calibration and measurement point selection. This is even more important when using partially unstable and semi-quantitative instruments or methods.

For *ED-XRF analysis* the measurement equipment was composed of an air-cooled X-ray tube with tungsten anode (0.6 mm focal spot, internal 3 mm Al filter, HV max. 50 kV and max. current 1 mA, although working conditions were 40 kV, 0.35 mA) and a cryogenic Germanium X-ray detector (EG&G ORTEC) with Beryllium window and a 195 eV energy resolution at the iron line.

Analysts (among authors) chose to abrade spots, up to 4 mm^2^, of the corrosion patina with an abrasive system before measurements in order to obtain data which was more representative of the bulk composition. Although micro-invasive, this technique allowed for measurements on many objects and produced a large number of element-concentration (%) data. As stated in literature, this method does not provide absolute, quantitative values but only relative percentages, although the use of reference materials with very similar alloy composition can produce semi-quantitative data acceptable for the purposes of this study.

The acquisition procedure was driven by a self-made computer software employing a simple graphic interface which helps in both the choice of acquisition parameters and the processing of spectra. The apparatus, built by two of the authors [33], was used *in situ*, placing it on a small movable wheelbarrow and the analysed point (position in which the exciting beam impinges on the bronze surface) was identified by a red laser pointer.

To convert files into a common format used as input by software for computation, the Calc package by LibreOffice (Libre Office 4, free from The Document Foundation, Germany) was employed; statistic computation was carried out by WinIdams (free by Unesco, FR), with MVSP (by Kowak Co., UK) and with Prof. Brereton’s add-in for Excel for multivariate analysis (free by Bristol Chemometrics, UK).

## Conclusions

This study was undertaken to answer a fundamental question posed by archaeologists: did ancient foundry-men intentionally change alloy composition of bronze objects in relation to their type of use?

As suggested by several authors, re-using objects and metal alloys is not only a modern-day practice. Experimental evidence shows that during the late Bronze Age, recasting of broken or damaged items was already common. Expert foundry-men had also discovered that heat can be saved by using broken objects instead of minerals (as suggested today by the “soft landing” idea, by Embodied Energy or Emergy Concept). In this case, multivariate analysis using an explorative and visual method, failed to identify homogeneous “clouds of objects” and seems to confirm the previously stated thesis. Physical properties of bronze objects were therefore probably obtained by thermo-mechanical treatments rather than intentionally changing alloys’ composition.

In more detail, although all the representations of the PC score space suggest a series of clusters, (shown in Table 9) none of the 5 grouping methods (see Fig. Y, AD, AH, AL, AP in the Additional file 1) suggest that there was a clear intention to change the percentages of the alloying elements for the investigated objects. The purpose of Table 9 is also to invite researchers to propose different grouping methods to verify whether there was a problem with variable definition in object grouping. However, our method does not seem to be erroneous as objects 1, 3 and 125, two axes and a casting residue, characterised by high Cu and low percentages of other elements, stay grouped in all projections.

**Table 9:** **Clouds of objects recognised in almost one of the Scores projections but not associated to any of the proposed groups**

**Timeline**	**code.n.**	**Description**	**Timeline**	**code.n.**	**Description**
eneolithic	44	axe, broaded borders	middle bronze	30979	nail
eneolithic	60898-2	axe, flat	middle bronze	66809-2	axe, raised edges, Nemi-Canterano type
final bronze	35780	skull	middle bronze	37633-2	nail
			middle bronze	1	axe Sezze type
final bronze, 1^ Fe	14228	leaf shape spearhead	late bronze	35790	brooch, violin bow shape
final bronze, 1^ Fe	14218-2	spearhead	late bronze	31212	brooch with eyelet
			late bronze	29920	needle
middle bronze	60904	axe, finned	late bronze	35810	brooch, arc and snake
middle bronze	36740-2	sword Cetona type	late bronze	94	ring
late bronze	60906	broad hatchet, Cuma type	late bronze	31667	needle
final bronze, 1^ Fe	14233-2	spearhead	final bronze	35807	foil tape
final bronze, 1^ Fe	67519-2	spearhead	final bronze	35794	brooch, fragment
			final bronze	35778	ring
antique bronze	60903-2	axe, raised edges	final bronze	31668	needle
middle bronze	60897-2	axe, finned	final bronze	31664	spring wire
final bronze, 1^ Fe	14228	leaf shape spearhead	final bronze	35844-2	earring
final bronze, 1^ Fe	14218-2	spearhead	final bronze	35842	needle
antique bronze	60893	axe, raised edges	middle bronze	30979	nail
antique bronze	60892-2	axe, raised edges	middle bronze	66809-2	axe, finned, Nemi-Canterano type
late bronze	14215-2	knife Bismantova type	middle bronze	37633-2	nail
			middle bronze	1	axe, raised edges, Sezze type
middle bronze	36740-2	sword Cetona type	late bronze	60906	axe Cuma type
final bronze, 1^ Fe	67519-2	spearhead	late bronze	35790	brooch, violin bow shape
			late bronze	31212	brooch with eyelet
antique bronze	60905-2	axe, raised edges	late bronze	29920	needle
late bronze	6	brooch with eyelet	late bronze	94	ring
final bronze, 1^ Fe	14220	spearhead	late bronze	35779	brooch, at elbow
			late bronze	31667	needle
middle bronze	60904	axe, finned	final bronze	35794	brooch, fragment
late bronze	67520-2	sickle Poggio Berni type	final bronze	35778	ring
final bronze, 1^ Fe	14233-2	spearhead	final bronze	31668	needle
final bronze, 1^ Fe	14229	spearhead	final bronze	31664	spring wire
final bronze, 1^ Fe	895	spearhead	final bronze	35844-2	earring
1^Fe	258-2	axe, finned, Ardea type	final bronze	35842	needle
1^Fe	361-2	sword sheath Guardiavomano type			

Only the first one, on the upper left, is composed by pure Cu objects of different age.

## Methods

### EDA-EFA

This data-set is considered a good example for the use of Exploratory Data Analysis or Exploratory Factor Analysis. After some computation no grouping is evident.

Contrarily, if a true classification method was used (a supervised method like Confirmatory Factor Analysis or LDA), data could be “adjusted” in order to obtain a desired classification, but with the associated risk of overfitting.

### Distributions

When cultural heritage objects are studied, a Gaussian or Gosset distribution cannot be considered obvious; this is shown well shown by the frequency distribution chart of elements contained in the Additional file 1. A non-parametric approach is therefore advisable, based on median, percentile, etc… for this reason the median absolute deviation from median (MAD) was used to study variations among measurements on the same object, while the percentiles were used for outlier checking.

MAD is robust in the presence of outliers, in contrast to the standard deviation which can be influenced by a single extreme value. Similarly, the interquartile range, or inter-percentile is robust versus outliers and can be used to detect an anomalous value.

**Figure 12:**
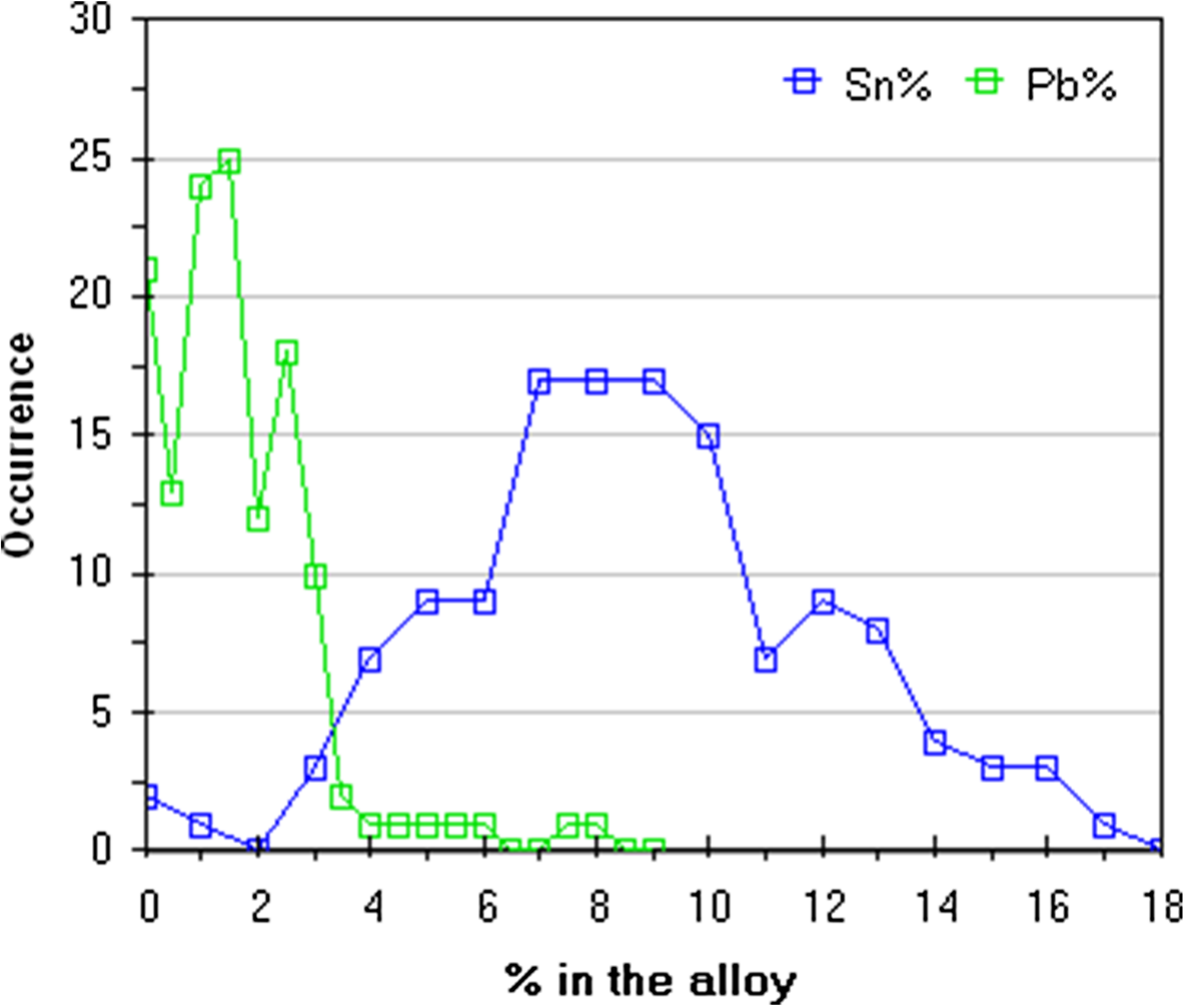
**Frequency distribution chart of the elements Sn and Pb in the 134 measured bronze objects.** As their distribution is not a normal one, it is difficult to identify outliers. However, many studies on the composition of ancient objects produce similar charts.

The importance of using non parametric methods for all our calculations can be demonstrated by Figure 12, showing the frequency distribution of Sn and Pb for the 134 objects – clearly not Gaussian distributions. Fig. C-J in the Additional file 1 show the frequency distribution of all 8 elements identified in the alloys.

### Scaling

As already stated, scaling is a necessary but treacherous pre-treatment of raw values. In any spectroscopic method, pre-treatment, like a simple derivation, can strongly improve information extraction from data. There are hundreds of references in this field, for example that of Joliffe, one of the fathers of PCA, studying the distortion effect [25]. As aforementioned, we decided to do a log-ratio scaling. For this purpose, the column of As was cut away from the raw data matrix and the remaining one was inserted in the MVSP software for computation. The software’s internal log-ratio function was used and successively PCA was calculated on the centred data.

## Endnote

^a^The importance of copper and bronze for manhood is so high that entire periods were termed after them, e.g. Copper Age = Chalcolithic = Eneolithic and later the Bronze Age.

## Additional file

Additional file 1: **Unfortunately a full chemometric analysis generates a large amount of data so for brevity additional material is presented in the Additional file** 1 **for further information.** a) a file in .XLS (2003 format) containing: the raw data for the 134 objects after median computation of the replicates in the engraved points and the data for the different methods of grouping; some sheets with frequency distribution charts for the elements and HLOC charts showing elements’ distribution in different manners; as well as the correlation and the covariance matrix for variables. b) many figures already cited in the text and identified by capital letters as belonging to the supplementary material (in high resolution); the long filenames should be explicative for their content.

## Abbreviations

XRF: ED-XRF: Energy dispersive - X-ray fluorescence analysis
PCA: Principal component analysis
HLOC: High-low-open-close chart
AAS: Atomic absorption spectroscopy
IC: Ion chromatography
MS: Mass spectroscopy
